# Racial and Socioeconomic Status among a Patient Population Presenting with Aneurysmal Subarachnoid Hemorrhage versus Unruptured Intracranial Aneurysm: A Single-Center Study

**DOI:** 10.3390/brainsci14040394

**Published:** 2024-04-18

**Authors:** Ashia M. Hackett, Christopher O. Adereti, Ariel P. Walker, Elsa Nico, Lea Scherschinski, Emmajane G. Rhodenhiser, Adam T. Eberle, Anant Naik, Juan P. Giraldo, Joelle N. Hartke, Redi Rahmani, Ethan A. Winkler, Joshua S. Catapano, Michael T. Lawton

**Affiliations:** 1 Department of Neurosurgery, Barrow Neurological Institute, St. Joseph’s Hospital and Medical Center, 350 W. Thomas Rd., Phoenix, AZ 85013, USAenico2@uic.edu (E.N.); lea.scherschinski@barrowneuro.org (L.S.); juanpedromed@gmail.com (J.P.G.); redir90@gmail.com (R.R.);; 2 Department of Neurosurgery, Lahey Hospital and Medical Center, Burlington, MA 01805, USA; christopheradereti@mail.rossmed.edu; 3 Department of Neurosurgery, Wayne State University School of Medicine, Detroit, MI 48201, USA

**Keywords:** aneurysmal subarachnoid hemorrhage, socioeconomic status, unruptured intracranial aneurysm

## Abstract

Racial and socioeconomic health disparities are well documented in the literature. This study examined patient demographics, including socioeconomic status (SES), among individuals presenting with aneurysmal subarachnoid hemorrhage (aSAH) and unruptured intracranial aneurysm (UIA) to identify factors associated with aSAH presentation. A retrospective assessment was conducted of all patients with aSAH and UIA who presented to a large-volume cerebrovascular center and underwent microsurgical treatment from January 2014 through July 2019. Race and ethnicity, insurance type, and SES data were collected for each patient. Comparative analysis of the aSAH and UIA groups was conducted. Logistic regression models were also employed to predict the likelihood of aSAH presentation based on demographic and socioeconomic factors. A total of 640 patients were included (aSAH group, 251; UIA group, 389). Significant associations were observed between race and ethnicity, SES, insurance type, and aneurysm rupture. Non-White race or ethnicity, lower SES, and having public or no insurance were associated with increased odds of aSAH presentation. The aSAH group had poorer functional outcomes and higher mortality rates than the UIA group. Patients who are non-White, have low SES, and have public or no insurance were disproportionately affected by aSAH, which is historically associated with poorer functional outcomes.

## 1. Introduction

There has been an increase in the literature highlighting the effect of social determinants on healthcare outcomes, particularly in relation to disparities experienced by different racial, ethnic, and socioeconomic groups [1,2,3,4]. Access to care and treatment outcomes for individuals with intracranial aneurysms vary significantly depending on their racial, ethnic, and socioeconomic backgrounds [5,6,7].

Aneurysmal subarachnoid hemorrhage (aSAH) is an urgent medical condition, associated with high rates of morbidity and mortality [6]. Based on the global incidence of aSAH at approximately 700,000 person-years and the prevalence of unruptured intracranial aneurysms (UIAs) at 3%, it is estimated that only around 0.3% of all UIAs rupture annually [8]. This suggests that the majority of UIAs are incidentally detected, particularly as a result of improved imaging techniques and more frequent use of neuroimaging for nonspecific symptoms like headache and vertigo [8,9]. However, despite these advancements, certain racial, ethnic, and socioeconomic groups continue to be disproportionately affected by the disparities in detecting and addressing UIAs [10]. Examining these disparities and related factors may lead to targeted interventions and strategies to mitigate disparities and improve overall healthcare outcomes for individuals affected by intracranial aneurysms.

This study aimed to analyze the demographics of patients who underwent microsurgical repair for unruptured and ruptured intracranial aneurysms. We hypothesized that aSAH, compared with UIA, would be disproportionately associated with non-White racial backgrounds, having public or no insurance, and having low socioeconomic status (SES).

## 2. Methods

### 2.1. Study Population

The study protocol was approved by the St. Joseph’s Hospital and Medical Center Institutional Review Board in Phoenix, Arizona. A detailed analysis of the demographics of the patient population admitted with aSAH to a high-volume cerebrovascular institution was performed. Patient demographic characteristics, aneurysm characteristics, and clinical data were obtained from electronic health records. All patients with an intracranial aneurysm who underwent microsurgical treatment between 1 January 2014 and 3 July 2019 at a single quaternary center were identified using retrospective research databases. Because of the retrospective nature of our study, the need for patient consent was waived. This analysis was deemed justifiable given the institution’s expertise in treating complex cerebrovascular cases, resulting in a substantial and relevant population suitable for analysis.

### 2.2. Data Collection

Patient demographic characteristics included sex, age, race and ethnicity, insurance type, and home address at the time of surgery. Race was defined according to the US Census Bureau as White, Black or African American (not mutually exclusive), American Indian or Alaska Native, Asian, Native Hawaiian or other Pacific Islander, or other race; ethnicity was defined as Hispanic or non-Hispanic [11]. Patients were dichotomized into 2 groups based on their self-identified race and ethnicity. Patients who identified as non-Hispanic for ethnicity and White as race were placed in the non-Hispanic White group, and all other patients were classified into the non-White group. Insurance type extracted from the medical chart was classified into the following groups: private (health maintenance organization or preferred provider organization), public (Medicaid or Medicare), military, other (e.g., insurance from a non-US provider or worker’s compensation), or uninsured. Patient home addresses were extracted from the electronic health record system. Using the University of Wisconsin School of Medicine’s Neighborhood Atlas website [12], an area deprivation index was obtained, as previously described by Knighton et al. [13] and Singh [14]. Using the area deprivation index, patients were assigned to 1 of 5 SES tiers, where tier 1 corresponded with the least disadvantaged and tier 5 with the most disadvantaged. Patients were identified as receiving treatment for either an aSAH or a UIA. aSAH was defined as bleeding into the subarachnoid space due to the rupture of an intracranial aneurysm, confirmed via the initial CT head, with the subsequent identification of the aneurysm on either CT angiography or digital subtraction angiography. Information on the prevalence of hypertension and cigarette smoking was collected because of the known association of these comorbidities with an elevated risk of aneurysm rupture [15,16]. Additional variables collected included Glasgow Coma Scale score at admission, aneurysm characteristics, and Charlson Comorbidity Index score. Primary outcomes included modified Rankin Scale (mRS) score, classified as good outcomes (0–2) or poor outcomes (3–6) at discharge; mRS score at follow-up; and mortality, which refers to death determined by neurological criteria.

### 2.3. Statistical Analysis

The statistical analysis was conducted using SPSS, version 26 (IBM Corporation; Armonk, NY, USA). Descriptive statistics were used to present the 2 patient subgroups admitted for the treatment of aSAH and UIA separately. The frequency and percentage or mean and SD were calculated. Comparative statistics were then performed to analyze and compare the aSAH and UIA populations with respect to the proportion of different patient subgroups. Comparisons were made using the Mann–Whitney *U* test for quantitative variables, the chi-squared test for categorical variables with an expected count of at least 5, and Fisher’s exact test for categorical variables with an expected count of less than 5. The Bonferroni correction post hoc test was applied to account for multiple testing comparisons.

A binary logistic regression model was employed to predict the clinical presentation of aSAH based on socioeconomic factors. The dependent variable was the clinical presentation of aSAH (e.g., presence or absence), whereas the independent variables included SES tier, race and ethnicity, and insurance type. Known risk factors for aSAH, such as age, comorbidities, and aneurysm characteristics, were included as covariates to control for potential confounding effects. Inverse probability of treatment weighting was applied to account for any potential selection bias. The results were reported using beta coefficients, *p*-values, odds ratios (ORs), and corresponding 95% CIs. Statistical significance was set at a threshold of 0.05. The sample size was determined by including all patients identified with an aneurysm who underwent microsurgical treatment during the period January 2014 to July 2019. The power analyses were performed post hoc, using the maximum sample. The statistical power of the test was estimated to be approximately 82.4%, indicating a high likelihood of correctly rejecting the null hypothesis if the true effect size is similar to the assumed value of Cohen’s *h* = 0.2, with a sample size of 640 and a significance level of 0.05.

## 3. Results

### 3.1. Description of Cohort

A total of 640 patients were included in the analysis, with 251 patients in the aSAH group and 389 patients in the UIA group. The results of the univariate analysis comparing patients with unruptured and ruptured aneurysms are displayed in Table 1.

The majority of the patients in both groups were female (285 of 389 [73.3%] in the UIA group and 172 of 251 [68.5%] in the aSAH group). There was no statistically significant difference in sex distribution between the UIA and aSAH groups (*p* = 0.74). A statistically significant difference in age was observed between the UIA and aSAH groups (*p* = 0.001). The mean (SD) age was 58.2 (12.3) years for the UIA group and 54.9 (13.3) years for the aSAH group, suggesting that individuals in the aSAH group tended to be younger.

### 3.2. Sociodemographic Differences

Race and ethnicity showed a highly significant association with aneurysm rupture (*p* < 0.001). The UIA group had a higher proportion of patients classified as White (272 of 389 [69.9%]) than the aSAH group (127 of 251 [50.6%]). SES tier also demonstrated a significant association with aneurysm rupture presentation (*p* = 0.004). The UIA group had a higher proportion of cases in SES tier 1 (83 of 389 [21.3%]) compared to the aSAH group (21 of 251 [12.4%]), whereas the aSAH group had a higher proportion in SES tier 5 (36 of 251 [14.3%]) compared with the UIA group (35 of 389 [9%]). When dichotomizing SES tiers into most disadvantaged (SES tiers 3–5) and least disadvantaged (SES tiers 1 and 2), there was a statistically significant difference in the proportion of least disadvantaged patients between the UIA and aSAH groups (*p* = 0.03). A higher proportion of patients were classified as least disadvantaged in the UIA group than in the aSAH group (168 of 389 [43.2%] vs. 84 of 251 [33.5%]).

Insurance type also showed a highly significant association with aneurysm rupture (*p* < 0.001). The UIA group had a higher proportion of patients with private insurance than the aSAH group (184 of 389 [47.3%] vs. 86 of 251 [34.3%]), whereas the aSAH group had a higher proportion of patients with public insurance (137 of 251 [54.6%]) compared with the UIA group (169 of 389 [43.4%]). Other variables also showed significant associations with aneurysm rupture presentation, including Glasgow Coma Scale score at admission, aneurysm location, family history of aSAH, smoking status, hypertension, and Charlson Comorbidity Index (Table 1).

The primary outcome variables assessed in this study were mRS score at discharge, mRS score at follow-up, and mortality rate (Table 2). At discharge, the aSAH group had significantly higher mean (SD) mRS scores at discharge (3.62 [1.44]) compared with the UIA group (2.19 [1.00]), indicating poorer functional outcomes (*p* < 0.001). During follow-up, the aSAH group continued to have significantly higher mean (SD) mRS scores (1.79 [1.31]) compared with the UIA group (1.34 [1.26]), indicating worse terminal functional outcomes (*p* < 0.001). Mortality was also significantly higher in the aSAH group (41 of 251 [16.3%]) compared with the UIA group (12 of 389 [3.1%]) (*p* < 0.001).

To further evaluate the associations between sociodemographic variables and the risk of developing aSAH, a binary logistic regression model was used (Table 3). The analysis accounted for multiple covariates, including sex, age, aneurysm size, comorbidities (hypertension, diabetes, and cardiovascular disease), family history of aneurysmal subarachnoid hemorrhage, Charlson Comorbidity Index, and smoking status. To mitigate potential bias, the model used inverse probability weighting.

Non-White patients had significantly higher odds of presenting with an aneurysmal rupture compared with White patients (odds ratio [OR] [95% CI] = 1.71 [1.23–2.36], *p* < 0.001). When compared with SES tier 1, patients in higher SES tiers had increased odds of a ruptured aneurysm at presentation (tier 2: OR [95% CI] = 2.24 [1.39–3.63], *p* = 0.001; tier 3: OR [95% CI] = 2.28 [1.43–3.62], *p* = 0.001; tier 4: OR [95% CI] = 2.25 [1.34–3.77], *p* = 0.002; tier 5: OR [95% CI] = 3.69 [2.07–6.58], *p* < 0.001). Regarding insurance type, both public coverage (public vs. private: OR [95% CI] = 1.55 [1.11–2.18], *p* = 0.01) and uninsured status (uninsured vs. private: OR [95% CI] = 2.93 [1.56–5.49], *p* = 0.001) were associated with higher odds of a ruptured presentation.

## 4. Discussion

This study examined patient demographics and outcomes among individuals who underwent microsurgical repair for both unruptured and ruptured intracranial aneurysms and found that aSAH, compared with UIA, was disproportionately associated with individuals from non-White racial backgrounds, those with public or no insurance, and those from moderately-to-highly disadvantaged SES groups. Significant associations between race and ethnicity and the presentation of aneurysm rupture were demonstrated. The proportion of patients categorized as White was higher in the unruptured intracranial aneurysm group than in the ruptured aneurysm group, whereas the proportion of non-White patients was higher in the ruptured intracranial aneurysm group than in the unruptured intracranial aneurysm group. Other studies on race and ethnicity have shown similar results regarding aSAH prevalence [6,17]. In a cross-sectional study using the National Inpatient Sample database, Kandregula et al. [10] found that, after adjusting for covariates, Black patients (OR [95% CI] = 0.637 [0.625–0.648]) and Hispanic patients (OR [95% CI] = 0.654 [0.641–0.667]) had lower odds of treatment compared with White patients. Interestingly, in the same study, multivariable regression analysis indicated that the odds of treatment had improved slightly over time among Black patients, whereas they had remained constant among Hispanic patients and other minorities [10]. Similar findings were reflected in a separate study, which reported that non-Hispanic Whites had the lowest proportion of aSAH diagnosis (30.91%; *p* < 0.001) [18].

In our analysis, UIA and aSAH risk had a high correlation with insurance type, with a higher percentage of patients in the UIA group than in the aSAH group having private insurance coverage and a higher percentage of patients in the aSAH group having public insurance coverage. Kandregula et al. [10] found that Medicare patients had higher odds of treatment than private patients, whereas Medicaid and uninsured patients had lower odds. In another study comparing the demographic characteristics of patients receiving treatment for UIAs and those treated for aSAH using the Vizient Clinical Database/Resource Manager database, the percentage of patients with Medicaid or without insurance was lower among patients admitted for UIA (21.8% vs. 24.5%; *p* = 0.006) [6].

Socioeconomic disparities were also evident. The unruptured group had a higher proportion of least-disadvantaged cases compared with the ruptured group. To our knowledge, this is the first study that determined socioeconomic status using The Neighborhood Atlas [12,19]. However, similar to our study, Rumalla et al. [7] analyzed other socioeconomic variables possibly influencing aSAH patient outcomes, including race, having a primary care physician at admission, family or caregiver support, a foreign language barrier, and primary payer status. The findings from this study revealed that, independent of follow-up duration, the presence of family or caregiver support was associated with a decreased likelihood of unfavorable outcomes (defined as mRS score > 2) during the last follow-up (OR [95% CI] = 0.14 [0.03–0.49], *p* = 0.004). Additionally, having an established primary care physician at the time of hospital admission was identified as the sole socioeconomic factor linked to a significantly lower risk of mortality (OR [95% CI] = 0.51 [0.26–0.98], *p* = 0.047), also independent of the follow-up duration [7].

Binary logistic regression analysis further supported the associations between sociodemographic factors and the risk of aneurysmal rupture. Non-White patients had significantly higher odds of presenting with an aneurysmal rupture, and higher SES tiers and being publicly insured or uninsured were also associated with increased odds of a ruptured presentation, highlighting the significant association between the risk of aneurysm rupture and SES, race and ethnicity, and insurance status. Our results also showed the effect of aneurysm rupture on functional outcomes and mortality rates. The aSAH group had significantly higher mRS scores at discharge and follow-up, indicating poorer functional outcomes, similar to other clinical outcomes of aSAH studies [20,21]. Moreover, the mortality rate was significantly higher in the aSAH group compared to the UIA group. Studies have indicated that healthcare professionals exhibit implicit racial and ethnic biases, and a correlation exists between these biases and clinical outcomes [22]. This relationship could potentially explain, in part, our findings.

Racial and socioeconomic health disparities are multifaceted, stemming from factors including limited healthcare access, existing comorbidities, and high-stress lifestyles. Restricted access to healthcare, whether due to factors such as insufficient insurance coverage, dependence on public insurance, transportation limitations, or geographical distance from medical facilities can result in latency of treatment, potentially amplifying the risk of aneurysmal rupture [23]. A study using the Nationwide Inpatient Sample database that examined patient demographics and treatment for UIA and aSAH from 2001 to 2009 found pronounced disparities, particularly among those younger than 65 years who do not qualify for Medicare insurance, underscoring the impact of insurance on these disparities. Nonetheless, disparities were evident even among insured individuals, as illustrated in a study by Brinjikji et al. [24], in which 81.6% of White patients covered by Medicare received treatment for UIAs, compared with only 4.7% of Black patients with the same insurance, highlighting that insurance status alone cannot account for these discrepancies [24,25]. Some data indicate that higher rates of SAH among Black and Hispanic populations are potentially linked to increased smoking and hypertension rates, factors well known to contribute to aneurysm rupture [26,27]. Additionally, high-stress lifestyles, which are more prevalent in lower socioeconomic and minority groups, in part as a result of financial insecurity and systemic racism, may further contribute to an increased risk of aSAH [28,29,30]. Further exploration of these factors can better inform efforts to address and mitigate health disparities experienced by certain demographic groups, with the aim of improving outcomes and providing equitable care for all individuals affected by intracranial aneurysms.

## 5. Limitations

The results of our research must be interpreted within the context of the study design. There are inherent limitations that accompany retrospective data collection. These include inferior levels of evidence when compared to prospective studies, increased potential for convenience sampling, and thus increased selection bias. However, we aimed to minimize these risks where possible in an attempt to provide accurate results. Another limitation of our study is that our sample population was taken exclusively from a single location that specializes in the treatment of intracranial aneurysms. This limits the external validity of our results such that they may not be reflective of those reported at nonspecialty centers. However, future studies comparing outcomes among specialty and nonspecialty centers can further highlight disparities that patients encounter across the neurosurgical community. Likewise, future multi-centered studies addressing our topic can add to the literature and strengthen the power of the findings reported in our study. As described earlier, our study includes a sample size of 640 patients, which limits the power and generalizability of our results. Again, the inclusion of larger prospective studies would help address these limitations and further add to the literature by allowing researchers to gather specific data that better underscore the disparities highlighted in our study while offering newer solutions that target these issues.

## 6. Conclusions

Non-White, publicly insured, and moderately-to-highly economically disadvantaged patients were more prevalent in the aSAH group than in the UIA group. Aneurysm rupture has a significant impact on functional outcomes and mortality rates, and our findings demonstrate that sociodemographic factors continue to influence health outcomes. Although studies have shown that implicit racial and ethnic biases have a relationship with health outcomes, further research exploring the underlying causes for, and solutions to, this disparity is necessary. This study should raise awareness of these issues; encourage the elimination of any institutional prejudice or biases that may contribute to these disparities; and promote healthcare equity, regardless of patient race and ethnicity, insurance status, or socioeconomic background.

## Figures and Tables

**Table 1:** Univariate analysis of demographic and clinical characteristics of patients who presented with UIA and aSAH.

Characteristic	UIA (*n* = 389)	aSAH (*n* = 251)	*p* Value
Sex			0.74
Female	285 (73.3)	172 (68.5)	
Male	104 (26.7)	79 (31.5)	
Age, mean (SD), years	58.2 (12.3)	54.9 (13.3)	0.001
Race and ethnicity			<0.001
Non-White	105 (27)	104 (41.4)	
White (non-Hispanic)	272 (69.9)	127 (50.6)	
Unspecified	12 (3.1)	20 (8)	
SES tier *			
Mean (SD)	2.72 (1.254)	3.02 (1.242)	0.004
Tier 1	83 (21.3)	31 (12.4)	
Tier 2	85 (21.9)	53 (21.1)	
Tier 3	106 (27.2)	72 (28.7)	
Tier 4	74 (19)	48 (19.1)	
Tier 5	35 (9)	36 (14.3)	
NR	6 (1.5)	11 (4.4)	
Insurance type			
Private	184 (47.3)	86 (34.3)	<0.001
Public	169 (43.4)	137 (54.6)	<0.001
Uninsured	15 (3.9)	22 (8.8)	<0.001
Other	18 (4.6)	6 (2.4)	
GCS score at admission			
Mean (SD)	14.89 (0.75)	11.39 (3.911)	<0.001
≥15	299 (76.9)	80 (31.9)	
<15	10 (3.5)	167 (66.5)	
NR	80 (20.6)	4 (1.6)	
Hypertension	240 (61.7)	159 (63.3)	0.35
Diabetes	60 (15.4)	31 (12.4)	0.15
CVD	3 (0.8)	57 (22.7)	<0.001
Family history of aSAH	94 (24.2)	17 (6.8)	<0.001
History of smoking	235 (60.4)	85 (33.9)	<0.001
CCI			
Mean (SD)	1.97 (1.492)	1.45 (1.363)	<0.001
0	83 (21.3)	77 (30.7)	
1	65 (16.7)	61 (24.3)	
2	106 (27.2)	57 (22.7)	
3	78 (20.1)	33 (13.1)	
4	39 (10)	13 (5.2)	
5	12 (3.1)	4 (1.6)	
6	4 (1)	1 (0.4)	
7	1 (0.3)	1 (0.4)	
8	1 (0.3)	0 (0)	
NR	0 (0)	4 (1.6)	

Data are no. (%) unless otherwise indicated. Abbreviations: aSAH, aneurysmal subarachnoid hemorrhage; CCI, Charlson Comorbidity Index; CVD, cardiovascular disease; GCS, Glasgow Coma Scale; NR, not reported; SES, socioeconomic status; UIA, unruptured intracranial aneurysm. * Pertains to the implications various economic and sociological factors (i.e., income, education, occupation, race/ethnicity) have on an individual’s or population’s overall access to economic resources, including health care.

**Table 2:** Univariate analysis comparing outcome variables of patients who presented with UIA and aSAH.

Variable	UIA (*n* = 389)	aSAH (*n* = 251)	*p* Value
mRS score at discharge			
Mean (SD)	2.19 (1.003)	3.62 (1.443)	<0.001
Good outcomes (0–2)	218 (56)	61 (24.3)	
Poor outcomes (3–6)	168 (43.2)	186 (74.1)	
NR	2 (0.5)	4 (1.5)	
mRS score at follow-up			
Mean (SD)	1.34 (1.253)	1.79 (1.314)	<0.001
Good outcomes (0–2)	318 (81.7)	124 (49.4)	
Poor outcomes (3–6)	46 (11.8)	44 (17.5)	
NR	25 (6.4)	83 (33.0)	
Mortality			
No. (%) of patients	12 (3.1)	41 (16.3)	<0.001
OR (95% CI)	0.03 (0.178)	0.16 (0.37)	

Data are no. (%) unless otherwise indicated. Abbreviations: aSAH, aneurysmal subarachnoid hemorrhage; mRS, modified Rankin Scale; NR, not reported; OR, odds ratio; UIA, unruptured intracranial aneurysm.

**Table 3:** Binary logistic regression model to predict ruptured aneurysm presentation.

Characteristic	*B*	*p* Value	OR (95% CI)
Female sex	−0.174	0.29	0.84 (0.611–1.156)
Age > 65 years	−0.013	0.96	0.988 (0.629–1.552)
Race		<0.001	
White vs. Non-White	0.534	0.001	1.706 (1.232–2.364)
White vs. unspecified	1.715	<0.001	5.558 (2.571–12.016)
Insurance		0.002	
Private vs. public	0.439	0.01	1.552 (1.107–2.175)
Private vs. uninsured	1.073	0.001	2.925 (1.557–5.494)
Private vs. other	−0.023	0.96	0.977 (0.436–2.192)
SES tier		<0.001	
Tier 1 vs. tier 2	0.808	0.001	2.244 (1.387–3.629)
Tier 1 vs. tier 3	0.822	0.001	2.275 (1.429–3.62)
Tier 1 vs. tier 4	0.809	0.002	2.245 (1.337–3.769)
Tier 1 vs. tier 5	1.306	<0.001	3.69 (2.071–6.577)

Abbreviations: *B*, beta coefficient; OR, odds ratio; SES, socioeconomic status.

## Data Availability

The data that support the findings in this study are available on request from the corresponding author.

## Abbreviations

ADI	area deprivation index
aSAH	aneurysmal subarachnoid hemorrhage
IBM	International Business Machines
mRS	modified Rankin Scale
OR	odds ratio
SES	socioeconomic status
UIA	unruptured intracranial aneurysm
